# Studies on the Protective Effect of Silybin Against Low-Dose Radiation-Induced Damage to the Immune System

**DOI:** 10.3390/ijms26125656

**Published:** 2025-06-12

**Authors:** Yu Zhang, Yanan Yu, Yue Gao, Lanfang Ma, Jie Xu, Lehan Ding, Hongling Zhao, Weixiang Hu, Kai Hou, Ping-Kun Zhou, Hua Guan

**Affiliations:** 1 School of Public Health, University of South China, Hengyang 421001, China; zhangyu18711452953@163.com (Y.Z.); 2571733009@aliyun.com (Y.Y.); 18591758230@163.com (Y.G.); baekhhan77@163.com (L.D.); 2 Beijing Key Laboratory for Radiobiology, Beijing Institute of Radiation Medicine, Beijing 100850, China; 2656103961@aliyun.com (L.M.); 18391862300@163.com (J.X.); 15735179923@163.com (H.Z.); 18273181567@163.com (W.H.); birm4th@163.com (P.-K.Z.); 3 School of Life Science, Hebei University, Baoding 071000, China; 4 College of Agronomy, Sichuan Agricultural University, Chengdu 611130, China; hking@sicau.edu.cn

**Keywords:** low-dose radiation, silybin, immune damage, protection

## Abstract

With growing public concern about the health effects of low-dose radiation, numerous studies have demonstrated that low-dose radiation can cause damage to the immune system, making intervention measures essential. This study investigated the protective effects of silybin against low-dose radiation-induced immune system damage and its underlying mechanisms at both the cellular and animal levels. At the cellular level, CCK-8 assays, ROS measurements, and RT-qPCR analysis revealed that silybin alleviated the reduction in RAW264.7 cell proliferation, intracellular ROS levels, and inflammatory cytokine expression following low-dose radiation exposure. At the animal level, comparative analyses of post-irradiation body weight, peripheral blood cell counts, immune organ coefficients, spleen HE/IHC staining, and spleen immune cell numbers demonstrated that silybin mitigated the radiation-induced decrease in body weight, reduction in peripheral blood leukocyte counts, inflammatory cell infiltration in the spleen, decline in spleen immune cell numbers, and increase in cGAS protein-positive cells. These findings indicate that silybin exerts protective effects against low-dose radiation-induced immune system damage, potentially by regulating the cGAS signaling pathway to reduce radiation-induced cellular injury, thereby enhancing its radioprotective properties.

## 1. Introduction

With the extensive use of radiation sources in fields such as medical treatment (e.g., CT scans, interventional procedures, gastrointestinal series, chest fluoroscopy), the nuclear industry (e.g., nuclear power generation, fuel extraction), and aerospace and aviation (e.g., cosmic rays, solar radiation), growing public concern has emerged regarding the health effects of low-dose radiation. According to the literature, low-dose radiation (LDR) is defined as exposure to external X-ray and gamma radiation with doses below 0.2 Gy and dose rates not exceeding 0.1 mGy/min, where the dose rate typically refers to the average exposure over a period of 1 h or more. In experimental contexts, LDR is generally considered to involve radiation doses below 0.5 Gy [1]. Substantial epidemiological evidence demonstrates that low-dose radiation exerts multidimensional impacts on the immune system, including functional impairments and molecular-level alterations: studies on atomic bomb survivors revealed thymic dysfunction and abnormalities in T cell-mediated cellular immunity [2,3]; residents in the Techa River nuclear contamination area exhibited persistent innate immune system modifications coupled with immune cell hyperactivation [4]; populations in Chernobyl-affected regions showed disrupted immune homeostasis [5]; significant alterations in immune pathway-related gene expression were observed in inhabitants of high natural background radiation areas [6].Given the cumulative detrimental effects of low-dose radiation on immune function, coupled with the current lack of targeted protective agents specifically addressing low-dose radiation-induced immune damage mechanisms, the development of such targeted therapeutics carries substantial clinical significance.

Currently, natural products with protective effects against low-dose radiation damage include polyphenolic epigallocatechin, apigenin, and caffeic acid phenethyl ester, which scavenge free radicals to exert antioxidant effects while providing cytoprotection [7,8,9,10]. It is hoped that by screening various natural products, one that effectively mitigates low-dose radiation-induced damage to the immune system can be obtained.This study aims to identify an effective natural compound from among three candidates: silybin, perilla proanthocyanidins, and pachymic acid A. Silybin, a natural flavonol isolated from *Silybum marianum*, exhibits a range of biological activities, including antioxidant [11], anticancer [11], and hepatoprotective effects [12]. Perilla proanthocyanidins are polyphenolic compounds derived from *Perilla frutescens*, known for their potent antioxidant bioactivity [13]. Poria acid A, a triterpenoid extracted from *Poria cocos*, possesses both antioxidant and anti-inflammatory bioactivities [14]. To date, no studies have investigated the protective effects of these three natural products against immune system damage induced by low-dose radiation, highlighting the necessity for such research.

In this study, RAW264.7 mouse macrophages and male BALB/c mice were used as research subjects. At the cellular level, the protective effects of natural products against low-dose radiation damage were detected via numerical changes in cell proliferative activity, reactive oxygen species, and mRNAs of inflammatory factors, and the protective effects and mechanism of silybin against immune system damage caused by low-dose radiation were further explored through animal experiments to provide experimental bases for the further research and development of silybin.

## 2. Results

### 2.1. RAW264.7 Cell Proliferation Viability Assay

**Figure 1:**
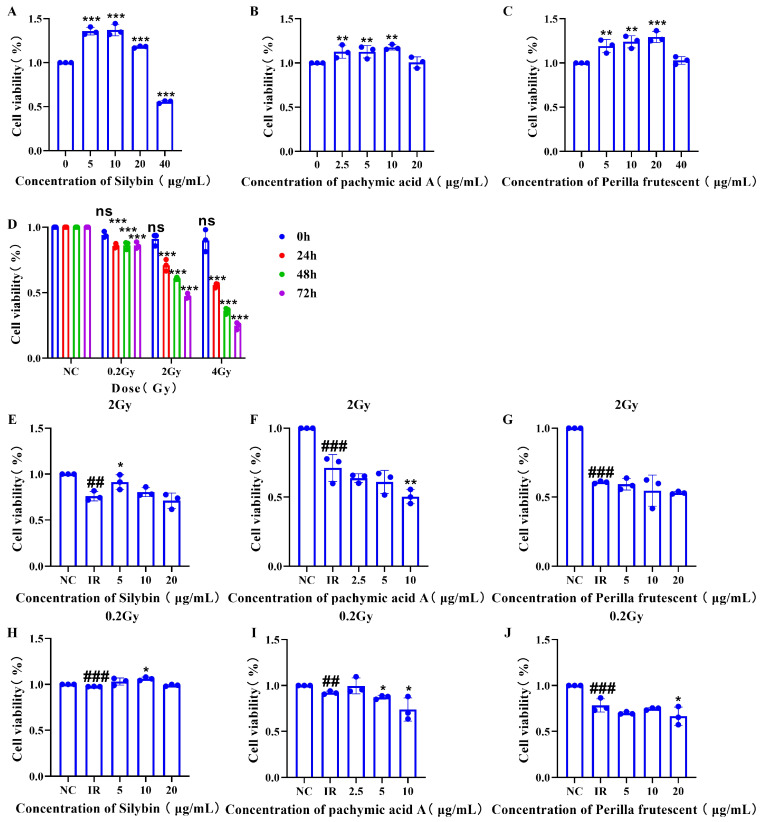
Proliferative viability of RAW264.7 cells after intervention with three natural products. (**A**–**C**) no irradiation, samples collected 48 h after intervention; (**D**) irradiation alone; (**E**–**G**) irradiation at 2 Gy, samples obtained 48 h post-irradiation; (**H**–**J**) irradiation at 0.2 Gy, samples acquired 48 h post-irradiation. The three natural products are silybin, pachymic acid A, and perilla proanthocyanidins. Data are presented as mean ± SEM. * *p* < 0.05; ** *p* < 0.01; *** *p* < 0.001; ## *p* < 0.01; ### *p* < 0.001; ns *p* > 0.05.

As shown in Figure 1A–C, under non-irradiated conditions, silybin, pachymic acid A, and perilla proanthocyanidin interventions enhanced RAW264.7 cell growth (*p* < 0.05). To determine whether these three natural products still have a pro-proliferative effect under irradiated conditions, we first irradiated RAW264.7 cells with 0.2 Gy, 2 Gy, or 4 Gy alone and collected the cells at 0, 24, 48, and 72 h for the CCK-8 assay to determine the appropriate irradiation dose and time points for sample collection.

As shown in Figure 1D, under simple irradiation, regardless of the irradiation dose, cell proliferation declined at 24, 48, and 72 h after irradiation (*p* < 0.05). We then determined 0.2 Gy and 2 Gy as the irradiation dose for the subsequent CCK-8 assay and 48 h after irradiation as the time point of sample collection for the subsequent CCK-8 assay.

As shown in Figure 1E–J, silybin promoted cell proliferation under both 2 Gy and 0.2 Gy irradiation conditions (*p* < 0.05).

These results indicate that silybin promotes cell proliferation more effectively than pachymic acid A or perilla proanthocyanidins.

### 2.2. Evaluation of the Antioxidant Activity of Natural Products

The CCK-8 results preliminarily revealed that silybin had a cell proliferation-promoting effect (*p* < 0.05), and other aspects of the impacts of the three natural products were further analyzed to determine which one to ultimately choose as an intervention for subsequent studies.

**Figure 2:**
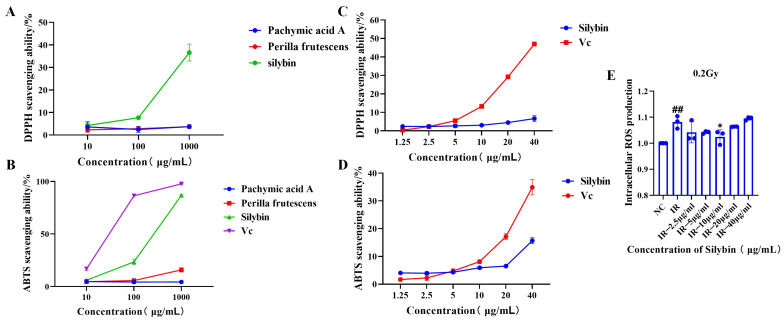
Evaluation of the antioxidant activity of natural products. (**A**,**B**) Three natural products—silybin, pachymic acid A, and perilla proanthocyanidins—and Vc (vitamin C) as positive controls; in vitro assay of the free radical scavenging ability of the natural products against DPPH and ABTS free radicals; (**C**,**D**) silybin and Vc as positive controls; in vitro assay of the DPPH and ABTS free radical scavenging ability of silybin; (**E**) flow assay of the effect of silybin on the ROS expression level in RAW264.7 cells 8 h after irradiation. Data are presented as mean ± SEM. * *p* < 0.05; ## *p* < 0.01.

As illustrated in Figure 2A,B, silybin demonstrated superior ABTS and DPPH free radical scavenging capacity compared to pachymic acid A and perilla proanthocyanidins at identical concentrations of 10, 100, and 1000 μg/mL. Consequently, silybin was selected for further investigation. As shown in Figure 2C,D, silybin exhibited scavenging activity against ABTS and DPPH free radicals at concentrations ranging from 1.25 to 40 μg/mL (1.25, 2.5, 5, 10, 20, and 40 μg/mL).

The above results indicate that silybin has antioxidant bioactivity, which can be further verified by its subsequent effects on cells.

The antioxidant effects of silybin after its action on cells are shown in Figure 2E. Under 0.2 Gy irradiation, 10 μg/mL silybin reduced the level of intracellular reactive oxygen species in RAW264.7 cells (*p* < 0.05), indicating the potential application value of silybin in attenuating oxidative stress and maintaining cellular homeostasis. We subsequently explored the anti-inflammatory effects of silybin.

### 2.3. Role of Silybin in the mRNA Levels of Inflammatory Factors in Irradiated RAW264.7 Cells

**Figure 3:**
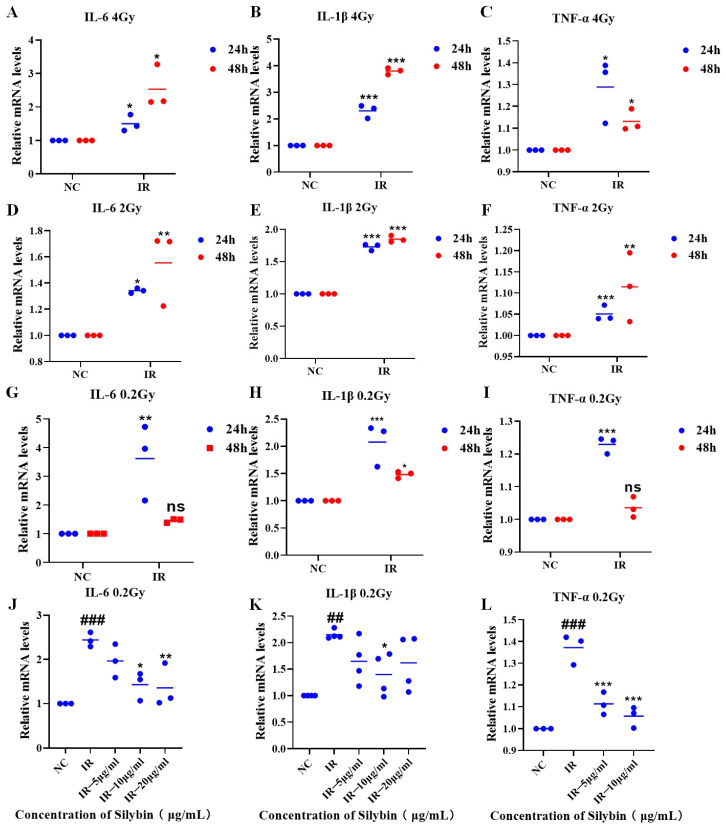
mRNA expression of inflammatory factors in RAW264.7 cells under irradiation conditions. (**A**–**I**) Irradiation alone: samples were collected 24 and 48 h after 4 Gy, 2 Gy, and 0.2 Gy irradiation, and the IL-6, IL-1β, and TNF-α mRNA levels in RAW264.7 cells were measured. (**J**–**L**) IL-6, IL-1β, and TNF-α mRNA levels in samples collected 24 h after 0.2 Gy irradiation combined with silybin intervention. Data are presented as mean ± SEM. ## *p* < 0.01; ### *p* < 0.001; * *p* < 0.05; ** *p* < 0.01; *** *p* < 0.001; ns *p* > 0.05.

As shown in Figure 3A–I, the expression of inflammatory factor mRNAs in RAW264.7 cells was increased (*p* < 0.05) at 24 and 48 h after irradiation with 4 Gy, 2 Gy, or 0.2 Gy, which was followed by charging a 24 h sample for the study under 0.2 Gy irradiation conditions.

As shown in Figure 3J–L, under 0.2 Gy irradiation conditions, silybin advanced intervention decreased the mRNA expression of IL-6, IL-1β, and TNF-α relative to that in the IR group (*p* < 0.05).

Therefore, silybin has a significant anti-inflammatory effect and reveals the potential mechanism by which it regulates the inflammatory response. Subsequently, the changes in several indices after the action of silybin on mice were investigated.

### 2.4. Effect of Silybin on the Body Weight of Irradiated Mice

**Figure 4:**
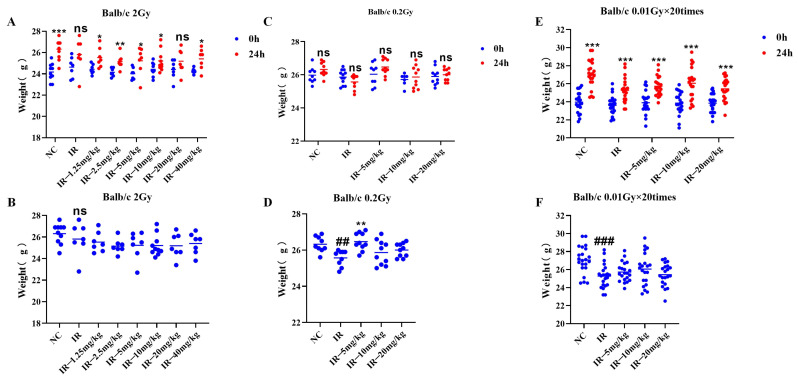
Changes in mouse body weight under different irradiation conditions caused by silybin: (**A**,**B**) single 2 Gy irradiation; (**C**,**D**) single 0.2 Gy irradiation; (**E**,**F**) multiple low-dose irradiation of 0.01 Gy × 20 times. Panels (**A**,**C**,**E**) show the mouse body weights before and after 24 h of irradiation, and panels (**B**,**D**,**F**) show the mouse body weights after 24 h of irradiation. For *n* ≥ 3, Data are presented as mean ± SEM. ## *p* < 0.01; ### *p* < 0.001; * *p* < 0.05; ** *p* < 0.01; *** *p* < 0.001; ns *p* > 0.05.

As shown in Figure 4A, the body weights of the mice in the different groups were greater than those of the mice prior to irradiation, except for those in the irradiated group (*p* < 0.05). As shown in Figure 4B, at 24 h following irradiation with 2 Gy, the body weights of the irradiated group of mice did not significantly decrease compared with those of the control group.

As shown in Figure 4C, the body weights of the mice in all the groups did not markedly differ from those before irradiation; as shown in Figure 4D, at 24 h following irradiation at 0.2 Gy, the body weights of the mice in the irradiated group were apparently lower than those in the control group, and the difference was statistically significant (*p* < 0.05), and those in the 0.2 Gy + 5 mg/kg group were greater than those in the irradiated group (*p* < 0.05).

As shown in Figure 4E, the body weights of all the groups increased relative to those before irradiation (*p* < 0.05). As shown in Figure 4F, at 24 h following irradiation at multiple low doses, the body weights of the irradiated group decreased relative to those of the control group (*p* < 0.05).

These three findings indicate that different irradiation conditions have different effects on mouse body weight.

### 2.5. Effect of Silybin on the Blood of Irradiated Mice

**Figure 5:**
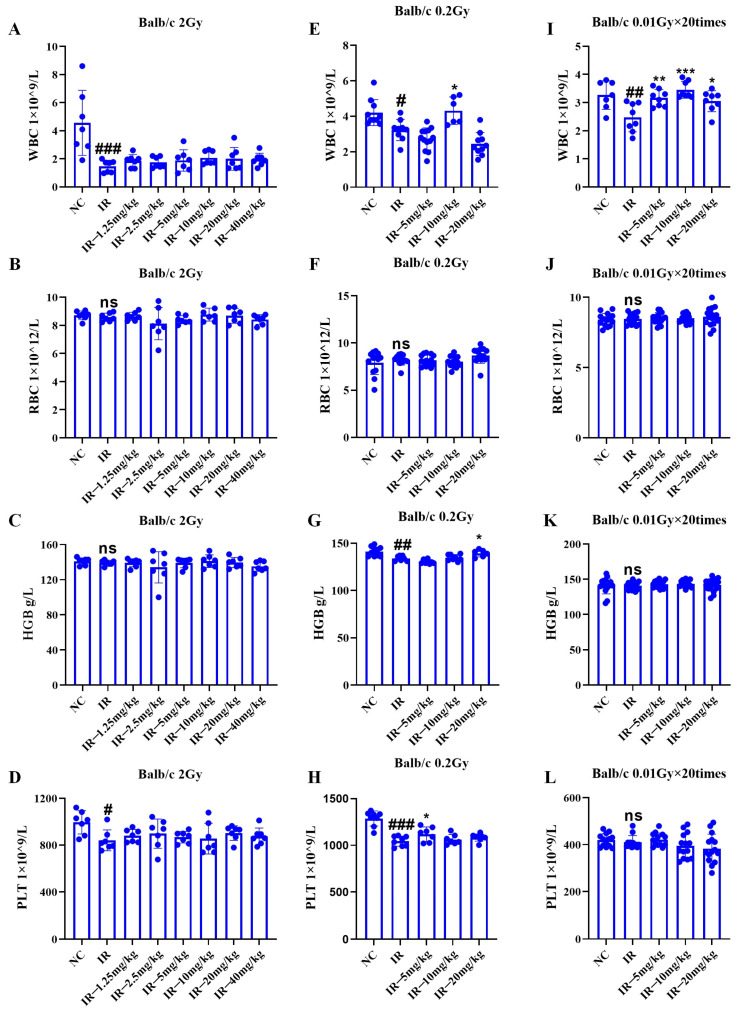
Changes in the blood profile of mice under different irradiation conditions caused by silybin: (**A**–**D**) single 2 Gy irradiation; (**E**–**H**) single 0.2 Gy irradiation; (**I**–**L**) multiple low-dose irradiation of 0.01 Gy × 20 times. *n* ≥ 3. Data are presented as mean ± SEM. # *p* < 0.05; ## *p* < 0.01; ### *p* < 0.001; * *p* < 0.05; ** *p* < 0.01; *** *p* < 0.001; ns *p* > 0.05.

As shown in Figure 5A–D, under 2 Gy irradiation conditions, the mouse platelet and leukocyte quantities decreased relative to those in the control group (*p* < 0.05); the number of leukocytes and platelets in the mice in the irradiated + administered group did not significantly increase compared with that in the irradiated group.

As shown in Figure 5E–H, under 0.2 Gy irradiation, the number of leukocytes, hemoglobin, and platelets in the mice decreased relative to those in the control group (*p* < 0.05); relative to those in the irradiation group, the number of leukocytes and platelets increased in both the irradiated + administered groups in the 1 group of mice (*p* < 0.05). Consequently, low-dose radiation can inhibit the hematopoietic system of mice, and this inhibitory effect is mitigated by intervention with silybin.

As shown in Figure 5I–L, under multiple low-dose irradiation conditions, the number of mouse leukocytes apparently decreased relative to that in the control group (*p* < 0.05); each irradiated + administered group had greater leukocyte count than the irradiated group (*p* < 0.05).

Consequently, low-dose radiation inhibited the mouse hematopoietic system, especially the number of leukocytes, and silybin intervention alleviated this inhibition.

### 2.6. Role of Silybin in the Immune Organ Coefficient in Irradiated Mice

**Figure 6:**
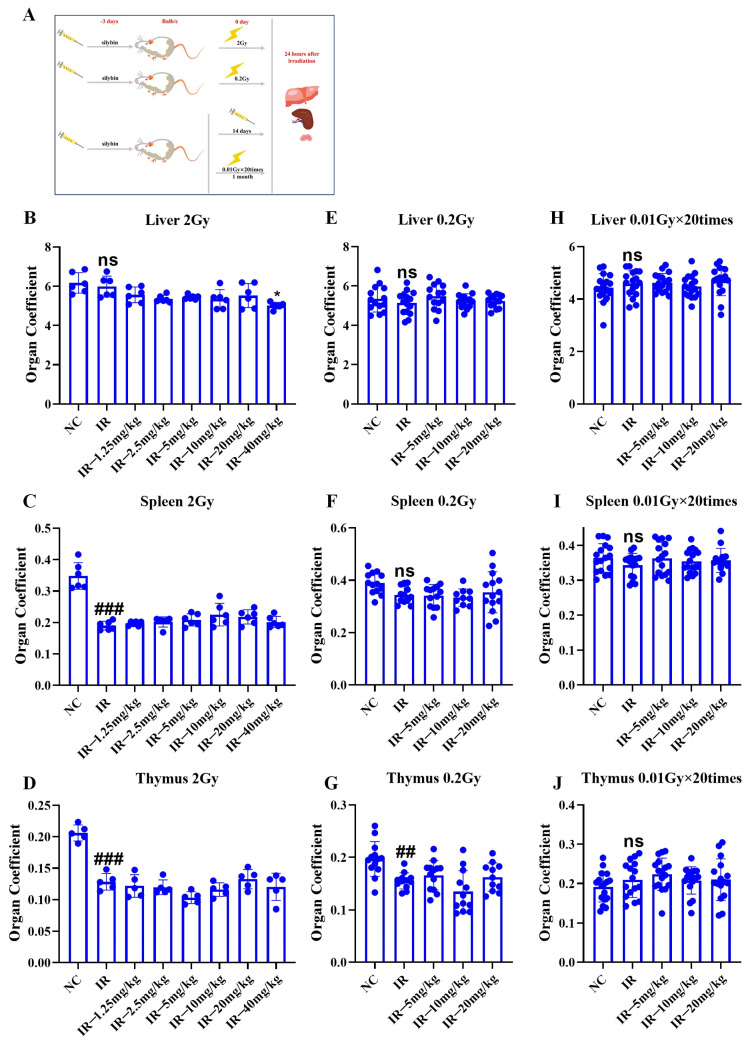
Changes in the organ coefficients of immune organs in mice under different irradiation conditions in response to silybin. (**A**) Flowchart of the experimental design for mice receiving tissues (liver, spleen, and thymus); (**B**–**D**) single 2 Gy irradiation; (**E**–**G**) single 0.2 Gy exposure; (**H**–**J**) multiple low-dose exposures of 0.01 Gy × 20 times. *n* ≥ 3. Data are presented as mean ± SEM. ## *p* < 0.01; ### *p* < 0.001; ns *p* > 0.05.

As shown in Figure 6A, the mice were given silybin 3 d before a single irradiation (2 Gy, 0.2 Gy), with a total of 3 d of intervention, and the last one was given 0.5 h before irradiation. The mice were irradiated multiple times (0.01 Gy × 20 times), with the intervention starting 3 d before irradiation, and administered daily at a fixed time for a total of 14 d. The mice received irradiation 5 times weekly (excluding weekends), totaling 20 sessions, and the samples were finally collected 24 h after the end of irradiation, and the livers, spleens, and thymuses of the mice were collected and weighed accordingly to calculate the organ coefficient.

As shown in Figure 6B–J, relative to the NC group, 2 Gy irradiation decreased the organ coefficients of both the spleen and the thymus (*p* < 0.05). Additionally, 0.2 Gy irradiation reduced the organ coefficient of the thymus (*p* < 0.05), while multiple low-dose irradiations did not significantly affect the organ coefficients of immune organs in mice. The above results indicate that single irradiation can cause damage to some immune organs in mice, and that silybin has no significant mitigating effect on such damage.

### 2.7. HE Staining of the Spleens of Mice Irradiated with Silybin

**Figure 7:**
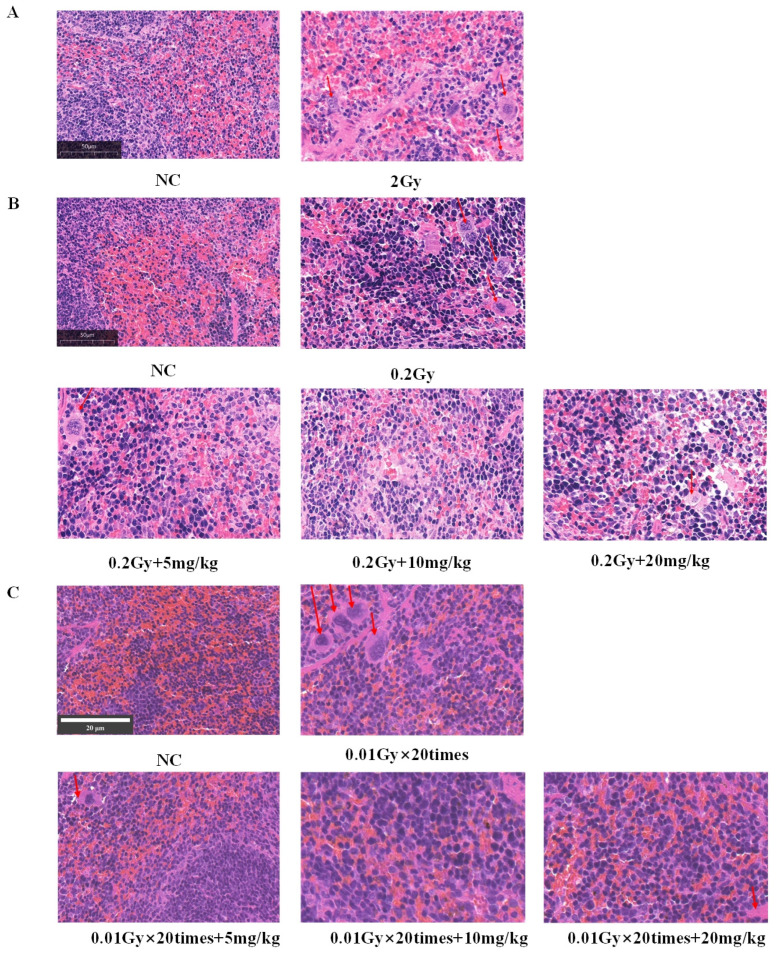
Silybin effects on mouse spleen HE staining under varied irradiation conditions: (**A**) 2 Gy; (**B**) 0.2 Gy, silybin intervention concentration 5/10/20 mg/kg; (**C**) 0.01 Gy × 20 times, silybin intervention concentration 5/10/20 mg/kg. The red arrows indicate inflammatory cell infiltration. Magnification 40×, scale 50 μm and 20 μm.

As illustrated in Figure 7A–C, under the three different irradiation conditions, the normal control (NC) group exhibited dense connective tissue in the splenic capsule, characterized by abundant smooth muscle fibers and elastic fibers of uniform thickness. Additionally, the capsular connective tissue extended into the spleen to form trabeculae, with no evident abnormalities observed. The splenic parenchyma comprised the white pulp, red pulp, and marginal zone. The white pulp consisted of periarterial lymphoid sheaths and lymphoid nodules, which displayed well-defined layers, were numerous, varied in size, and irregular in shape. The red pulp was located beneath the capsule. The red medulla oblongata is distributed in the pericardium, around the trabeculae, and the outer part of the marginal zone of the white medulla oblongata and consists of the splenic cord and the splenic blood sinus. Compared with the NC group, all the IR groups presented different degrees of inflammatory cell infiltration. The degree of inflammatory cell infiltration was attenuated in the irradiation intervention group compared with the IR group.

These results suggest that silybin has a certain mitigating effect on the radiation-induced inflammatory response of the spleen and may exert its protective effect by inhibiting the inflammatory response or protecting the splenic tissue under different irradiation conditions via the action of silybin.

### 2.8. Role of Silybin in the Number of Splenic Immune Cells in Irradiated Mice

**Figure 8:**
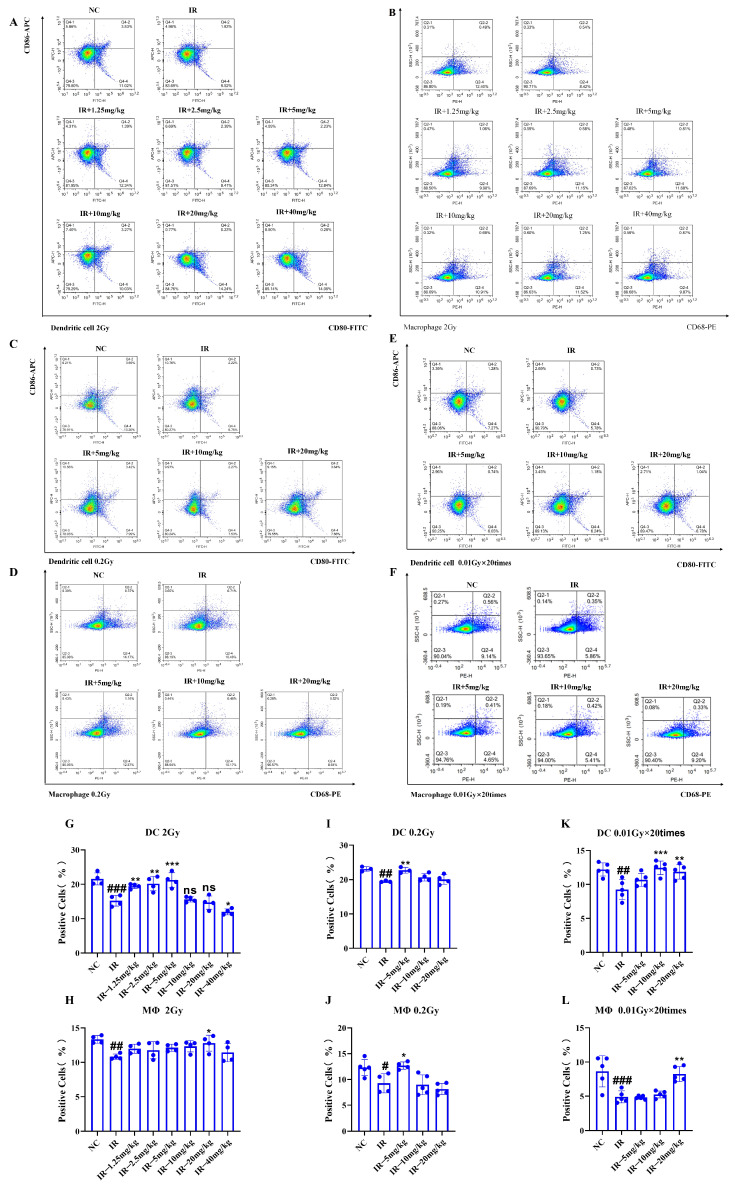
Alterations in the number of splenic immune cells in mice subjected to distinct irradiation conditions caused by silybin: (**A**,**B**,**G**,**H**) 2 Gy; (**C**,**D**,**I**,**J**) 0.2 Gy; (**E**,**F**,**K**,**L**) 0.01 Gy × 20 times. *n* ≥ 3. Data are presented as mean ± SEM. # *p* < 0.05; ## *p* < 0.01; ### *p* < 0.001; * *p* < 0.05; ** *p* < 0.01; *** *p* < 0.001; ns *p* > 0.05.

As shown in Figure 8A–L, under different irradiation conditions, the numbers of dendritic cells (DCs) and macrophages in the IR group decreased in comparison with those in the NC group (*p* < 0.05). However, following the silybin intervention, the number of DCs and macrophages increased (*p* < 0.05).

Therefore, silybin efficiently alleviates radiation-induced immunocytopenia and may exert its protective effect by promoting the proliferation and survival of immune cells or inhibiting radiation-induced apoptosis. These results suggest that silybin has potential application value in improving radiation-induced immunosuppression.

### 2.9. Results of IHC Staining of the Spleens of Mice Irradiated with Silybin

**Figure 9:**
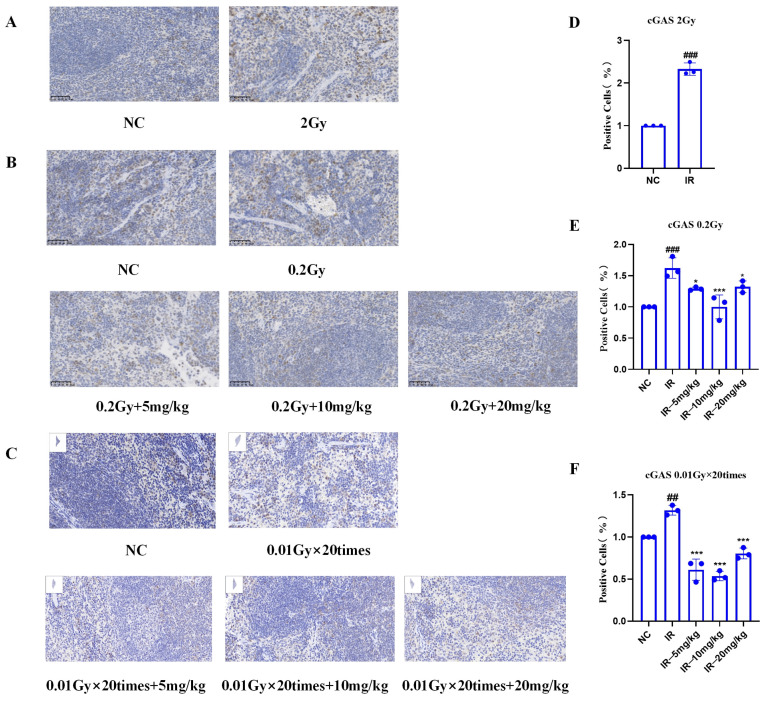
Immunohistochemistry of splenic cGAS proteins in mice subjected to different irradiation conditions via silybin: (**A**,**D**) 2 Gy; (**B**,**E**) 0.2 Gy; (**C**,**F**) 0.01 Gy × 20 times. Magnification 40×, scale 50 μm and 20 μm. *n* ≥ 3. Data are presented as mean ± SEM. ## *p* < 0.01; ### *p* < 0.001; * *p* < 0.05; *** *p* < 0.001.

As shown in Figure 9A–C, under different irradiation conditions, the IR group presented significant positive cell expression relative to the NC group, indicating the role of radiation in increasing the cGAS protein level. However, fewer positive cells (*p* < 0.05) were detected following silybin intervention at 0.2 Gy and 0.01 Gy × 20 Gy, suggesting that the natural product effectively inhibited radiation-induced cGAS protein expression.

These results suggest that silybin may play a protective role against radiation by attenuating radiation-induced cellular damage through regulation of the cGAS signaling pathway.

## 3. Discussion

Exposure to low-dose radiation has been associated with an elevated risk of cardiovascular disorders [15], cataracts [16], and thyroid cancer [17]. Low-dose radiation can also affect the immune system. Epidemiologic cohort studies have shown [3,18,19] that low-dose radiation-induced T-cell immune senescence leads to decreased T-cell-mediated immunity. Heylmann reported [20] that monocytes, DCs, regulatory T cells, and macrophages exhibited increased resistance to radiation. Few studies have focused on innate immune cells, which, like natural killer cells, macrophages, and DCs, play important roles in mediating radiation-related immune responses [21]. Therefore, there are relatively few studies on how low-dose radiation affects dendritic cells and macrophages to modulate immune responses. In the present study, we found that the number of splenic dendritic cells and macrophages that can be caused by low-dose radiation was significantly reduced (*p* < 0.05), which coincides with the findings of Bogdandi et al. [22,23], who utilized a mouse model in which a single dose of low-dose radiation (100 mGy) was used to induce a mild and transient reduction in splenocyte subpopulations.

Currently, several interventions have demonstrated efficacy in protecting against radiation-induced damage to the immune system. Malhotra [24] employed N-acetyl tryptophan glucoside (NATG), capitalizing on its anti-inflammatory properties to confer protection. Similarly, Sanhu Zhao [25] utilized hydrogen-rich saline, leveraging its antioxidant capabilities to mitigate such immune system damage. Adhikari [26] used silybin to protect against hematopoietic and immune system damage caused by 9 Gy γ-radiation via its antioxidant properties; Aihong Mao [27] used melatonin to protect against immune system damage caused by carbon ion radiation in mice through its antioxidant and antiapoptotic properties. The above products protect against radiation-induced immune system injury through oxidation, inflammation, and apoptosis. Nonetheless, few studies concerning interventions for low-dose radiation-induced immune system injury exist. Our study fills this gap. Our study revealed that silybin could exert anti-inflammatory and antioxidant effects, protect against peripheral blood leukocyte injury, reduce the organ coefficient of immune organs, and reduce dendritic cell and macrophage counts in the mouse spleen under low-dose irradiation conditions. We will continue to explore the possible protective mechanisms of silybin.

Studies have shown that silybin inhibits O-acetylglucosamine glycosylation, further reducing NF-κB and its downstream overexpression of TNF-α and IL-6 and inducing nitric oxide synthase type I (iNOS), which attenuates inflammatory responses [28]. It also activates the nuclear factor-related factor 2 (Nrf2) pathway, directly reducing ROS levels [29]. Moreover, innate immunity is associated with the cGAS-STING signaling pathway [30]. Our mouse spleen immunohistochemistry results revealed that silybin further protects against radiation by modulating the cGAS signaling pathway to attenuate radiation-induced cellular damage. However, its specific mechanism needs to be studied more thoroughly.

In conclusion, low-dose radiation may impact the immune system in multiple ways, including the suppression of immune cell activity, damage to immune organs, and disruption of immune factors [31]. These effects can contribute to reduced immune function and an elevated risk of infection and disease. Conversely, intervention with silybin has demonstrated efficacy in alleviating immune damage induced by low-dose radiation, offering a promising approach for radiation protection. A limitation of this study is the insufficient elucidation of the specific mechanisms through which silybin confers its protective effects against immune injury. With continued investigation, natural product-based strategies for radiation protection are expected to play a significant role in protecting human health.

## 4. Materials and Methods

### 4.1. Natural Product

The three natural products (silybin, pachymic acid A, and perilla proanthocyanidins) were kindly provided by Associate Prof. Kai Hou from the College of Agronomy, Sichuan Agricultural University.

### 4.2. Cells

Our laboratory produced RAW264.7 cells, which were initially sourced from Wuhan Pricella Biotechnology Co., Ltd. (Wuhan, China). They were cultivated in high-sugar (DMEM) supplemented with 10% fetal bovine serum (FBS) and 1% double antibody at 37 °C, 5% CO_2_, and saturated humidity. The 90% confluent cells were divided into the normal control group, irradiation group, and irradiation intervention group; the intervention was performed 0.5 h before irradiation; the irradiation doses were 2 Gy and 0.2 Gy; and the samples were collected 24 h and 48 h after irradiation.

### 4.3. Cellular Intervention Natural Product Formulation

Silybin, perilla proanthocyanidins, and pachymic acid A were all mixed with a 40 mg/mL master mixture in DMSO, after which they were stored in portions at −20 °C. The final concentration of the intervention was diluted 1000-fold with a complete culture medium.

### 4.4. Cell Proliferation Viability Assay

To prepare the cell suspension, cells were counted and inoculated into 96-well plates at 3000 cells/100 μL/well, 3 replicate wells were set up, the samples were incubated at 37 °C for 12 h in an incubator, the old medium was discarded, 100 μL of different concentrations of the samples to be tested were added to each group, the samples were incubated at 37 °C for 0, 24, 48, or 72 h in the incubator, the old medium was discarded, and 10% CCK-8 (MeilunBio, Liaoning, China) reagent (100 μL) was added to each well. The bio-solution was poured into every well, followed by gentle tapping of the plate after the addition of reagents to help mix, and then, the mixture was placed in the incubator at 37 °C for 2 h. A microplate reader (Molecular Devices, SpectraMax i3x, Shanghai, China) was used to detect the absorbance at 450 nm.

### 4.5. Free Radical Scavenging Capacity of Natural Products

The spectrophotometer/anemometer was preheated for more than 30 min, the wavelength was adjusted to 405 nm, and the Vc as well as the three natural products were set to 10, 100, and 1000 μg/mL (100 μL each), which strictly followed the operating procedures of the instruction manual of the free radical scavenging ability detection kits of ABTS and DPPH (Beijing Solarbio Science & Technology Co., Ltd., Beijing, China) sample addition.

### 4.6. Cellular Reactive Oxygen Species Assay

Serum-free medium was added to dilute DCFH-DA (Shanghai Beyotime Biotechnology Co., Ltd., Shanghai, China) at a ratio of 1:1000 to reach a final concentration of 10 M. The cell culture medium was removed, and 1 mL of diluted DCFH-DA (60 mm cell culture dish) was added for 20 min of incubation at 37 °C within the incubator. The cells were rinsed three times with a serum-free medium to remove the DCFH-DA that did not enter the cells. DCFH-DA inside cells. Trypsin-digested cells were transferred into 1.5-mL EP tubes at 3000 r/min and centrifuged for a 5 min period, followed by resuspension in PBS and flow (Aisen Biological (Hangzhou) Co., Hangzhou, China)

### 4.7. Real-Time Fluorescence Quantitative PCR Assay

In a 60 mm cell culture dish, 1 mL of TsingZol (BeijingTsingke Biotech Co., Ltd., Beijing, China) was added and mixed well, then 200 μL of chloroform was added, and the mixture was inverted and blended sufficiently. The mixture was incubated for 5 min at ambient temperature, followed by 15 min of centrifugation (4 °C, 12,000 r/min). The supernatants were collected in another centrifuge tube, and the same amount of isopropanol (Sinopharm Chemical Reagent Co., Ltd., Beijing, China) was added and mixed well. The mixture was incubated at 4 °C for 15 min at 4 °C and 12,000 r/min, and the supernatants were discarded. The mixture was centrifuged at 12,000 r/min for 15 min, the supernatant was discarded, 75% ethanol was added to wash it at 4 °C and 8000 r/min, the mixture was centrifuged for 5 min, the supernatant was discarded, the mixture was discarded, the RNA precipitate was blow-dried on an ultraclean table, the precipitate was dissolved in RNase-free water, the concentration of RNA was tested via nucleic acid and protein quantification, and then, the process was performed in strict accordance with the Reverse Transcription Kit (Nanjing Novozymes Biotechnology Co., Ltd., Nanjing, China) and qPCR kit (BeijingTsingke Biotech Co., Ltd., Beijing, China). The primers listed Table 1 were utilized. Primer sequences of the target gene were searched on the NCBI website, and the synthesis of the relevant qPCR primers was entrusted to Beijing Tsingke Biotechnology Co., Ltd. (Beijing, China).

### 4.8. Animals

SPF-grade male BALB/c mice that were 6–8 weeks old (20–25 g) were obtained from Beijing Specific Technology Co., Ltd. (Beijing, China), Animal License No. SCXK (Beijing 2024-0001), and their body weights, peripheral blood hematology, immune organ coefficients, splenic HE/IHC staining, and splenic macrophage/dendritic cell analysis were tested. The animals were housed at 22–24 °C and 52%~58% humidity. All animal experimental protocols were approved by the Experimental Animal Ethics Committee of the Military Medical Research Institute (Animal Ethics No: IACUC-DWZX-2021–540) and were conducted in accordance with established guidelines for animal experimentation. Following a one-week acclimatization period, the animals were randomly assigned to the normal control, irradiation, and irradiation intervention groups, with 20 mice in each group, totaling 300 mice (including single-dose 2 Gy and 0.2 Gy irradiation, as well as 0.01 Gy × 20 fractionated irradiation).

### 4.9. Radiation Exposure

The ^60^Coγ radiation source was obtained from the Institute of Radiation Medicine of the Military Medical Research Institute. Irradiation was conducted at dose rates of 0.6896 Gy/min for 2 Gy, 0.2473 Gy/min for 0.2 Gy, and 0.0258 Gy/min for 0.01 Gy. Single-dose exposures included 2 Gy and 0.2 Gy, while the multiple-dose regimen consisted of 0.01 Gy administered 20 times over the course of one week (five irradiations per week). Sample collection was performed 24 h after exposure for both single and multiple irradiation protocols.

### 4.10. Animal Intervention Natural Product Formulation

Silybin was prepared in master batches of 1.25, 2.5, 5, 10, 20, or 40 mg/kg in DMSO and then stored at −20 °C in portions. For use, the mixture was diluted 10-fold with the appropriate cosolvent to the desired dry prefinal concentration. Cosolvents: 10% DMSO, 45% physiological saline, 40% PEG300, and 5% Tween-80. Intervention mode (gavage): 0.5 h before irradiation, 3 d before irradiation, a total of 3 d of single low-dose radiation, and 11 d after multiple low-dose radiation, for a total of 14 d of gavage.

### 4.11. Weight

The mice were weighed 24 h before and 24 h after irradiation.

### 4.12. Peripheral Blood

Blood samples were collected from the retro-orbital sinus of mice 24 h post-irradiation. Hematological parameters, including red blood cell count (RBC), white blood cell count (WBC), platelet count (PLT), and hemoglobin concentration (HGB), were measured using a fully automated blood cell analyzer (NIHON KOHEDN MEK-300, Shanghai Kohden Medical Electronic Instrument Corp, China).

### 4.13. Immune Organ Coefficient

The livers, spleens, and thymuses of the mice were weighed at 24 h post-irradiation to calculate the organ index.

### 4.14. HE Staining of the Spleen

The spleens of the mice were removed at 24 h post-irradiation, placed in 4% tissue fixative at room temperature for 24 h, desiccated, dipped in wax, embedded in paraffin, and sectioned. The sections were subsequently stained after a series of pretreatments, such as dewaxing. Hematoxylin staining was performed first, followed by eosin staining, and finally, the sections were dehydrated and sealed. At this point, the HE-stained sections were prepared for scanning.

### 4.15. Flow Analysis of Splenic Immune Cells

Freshly collected mouse spleen tissues from each group were obtained, and a 70 μM filter was subsequently used to grind them into a single-cell suspension. After 5 min of centrifugation (1500 r/min), the supernatants were removed, and the cells were resuspended by adding 4 mL of PBS. This operation was conducted two times, and PBS (4 mL) was added for cell resuspension. Then, 300 μL resuspension was added to 3 μL of CD16/32 (Biolegend Beijing Biotech Co., Ltd., Beijing, China) to block the nonspecific Fc receptor. Subsequently, CD80, CD86, and CD68 antibodies (Biolegend Beijing Biotechnology Co., Ltd., Beijing, China) were added, the mixture was incubated at 4 °C for 1 h, 3 volumes of erythrocyte lysate (Tianjin Hao Yang Biological Products Science and Technology Co., Ltd., Tianjin, China) was added, the mixture was lysed for 15 min at room temperature, and then the cells were rinsed with PBS twice (1500 r/min, 5 min each). After the supernatants were discarded, the cells were washed with PBS twice and then washed with PBS for 5 min. The cells were washed twice with PBS at 1500 r/min for 5 min, the supernatant was discarded, and the cells were resuspended in 300 μL of PBS.

### 4.16. IHC Staining of the Spleen

Immunohistochemical pathology sections were obtained by a series of processes, such as paraffin sectioning for dewaxing, antigen retrieval, endogenous peroxidase blocking, serum blocking, primary antibody-adding secondary antibody incubation, DAB chromatography, nuclear replication, dehydration, and sealing. After being scanned by the section scanner, 10 fields of view of each slice were randomly selected separately, and 3 fields of view were randomly selected for ImageJ quantitative analysis. The presence of tan and brownish-yellow granules in the nuclei of splenic tissue cells was positive, whereas the absence of coloration in the nuclei, or very light coloration, was negative.

### 4.17. Statistical Methods

The experimental data are presented as means ± standard deviations. GraphPad Prism 9.0 software was used for analysis. The immunohistochemical results were quantitatively analyzed using ImageJ1 1.54f software (ImageJ bundled with 64-bit Java 8). One-way *t*-tests were used to compare the normal control and irradiation groups, whereas one-way ANOVA was used to compare the irradiation and irradiation intervention groups; *p* < 0.05 represented statistical significance.

## Figures and Tables

**Table 1:** Primer sequences.

Primer Name	Primer Sequence (5′-3′)
*β-actin*	F: GCAAGCAGGAGTACGATGAGT
	R: AAAACGCAGCTCAGTAACAGTC
*IL-6*	F: ACAACCACGGCCTTCCCTA
	R: CATTTCCACGATTTCCCAGA
*IL-1β*	F: GGGCTGGACTGTTTCTAATGC
	R: CTTGTGACCCTGAGCGACC
*TNF-α*	F: TTAGAAAGGGGATTATGGCTCA
	R: TTTGCAGAACTCAGGAATGGAC

## Data Availability

Data is contained within the article.

## Abbreviations

The following abbreviations are used in this manuscript:

CD68	cytoplasmic glycoprotein
CD80	T-lymphocyte activation antigen
CD86	T-lymphocyte activation antigen
cGAS	cyclic GMP-AMP synthase
HE	hematoxylin–eosin staining
h	hour
LDR	low dose radiation
IHC	immunohistochemistry
IR	irradiation group
NC	negative control group
ROS	reactive oxygen species
Vc	vitamin C
